# Role of Enzymatic Reactions in Meat Processing and Use of Emerging Technologies for Process Intensification

**DOI:** 10.3390/foods12101940

**Published:** 2023-05-10

**Authors:** Blanca Abril, Ricard Bou, Jose V. García-Pérez, Jose Benedito

**Affiliations:** 1 Department of Food Technology, Universitat Politècnica de València, 46022 Valencia, Spain; 2 Food Safety and Functionality Program, Institut de Recerca i Tecnologia Agroalimentàries (IRTA, Monells, Girona), 17121 Girona, Spain

**Keywords:** enzymes, enzyme reaction, meat, meat processing, emerging technologies, intensification

## Abstract

Meat processing involves different transformations in the animal muscle after slaughtering, which results in changes in tenderness, aroma and colour, determining the quality of the final meat product. Enzymatic glycolysis, proteolysis and lipolysis play a key role in the conversion of muscle into meat. The accurate control of enzymatic reactions in meat muscle is complicated due to the numerous influential factors, as well as its low reaction rate. Moreover, exogenous enzymes are also used in the meat industry to produce restructured products (transglutaminase), to obtain bioactive peptides (peptides with antioxidant, antihypertensive and gastrointestinal activity) and to promote meat tenderization (papain, bromelain, ficin, zingibain, cucumisin and actinidin). Emerging technologies, such as ultrasound (US), pulsed electric fields (PEF), moderate electric fields (MEF), high-pressure processing (HPP) or supercritical CO_2_ (SC-CO_2_), have been used to intensify enzymatic reactions in different food applications. This review aims to provide an overview of the enzymatic reactions taking place during the processing of meat products, how they could be intensified by using emerging technologies and envisage potential applications.

## 1. Introduction

Meat aging is a combination of transformations that originate in the animal’s muscle after slaughter, resulting in changes in colour, tenderness and aroma [1]. The biochemical processes that occur during meat aging are mainly caused by endogenous enzymes, leading to glycolysis, proteolysis and lipolysis. In the glycolysis reactions, glucose is metabolised to produce lactic acid, which lowers muscle pH and depletes the energy reserves (ATP). The energy depletion leads to the degradation of myofibrillar proteins by the action of endopeptidases and the action of exopeptidases. Endogenous proteases (calpains, cathepsins and calpastatin) play a crucial role in the proteolysis of meat; however, exogenous proteases (peptidylpeptidases, aminopeptidases and carboxypeptidases) secreted from microorganisms involved in meat fermentation also contribute to increase the concentration of peptides and amino acids [2]. Another reaction that takes place during the meat aging is the lipolysis in the muscle and the adipose tissue [3]. 

The products resulting from the degradation of proteins and lipids are precursors of the characteristic flavour and aroma of the meat and meat products [4,5,6]. In addition, the fragmentation of the myofibrils also leads to changes in texture that lead to meat softening [7]. Therefore, post mortem changes in the muscle, which affect the organoleptic properties of meat, are mostly related to the action of enzymes. Moreover, the enzymatic reactions that take place in the transformation of muscle into meat occur at relatively low reaction rates and are affected by numerous intrinsic (animal breed, age or feeding) and extrinsic factors (such as temperature, animal welfare, transport, stress, etc.). On the other hand, exogenous enzymes are also used in the meat industry, mainly to produce restructured meat, obtain bioactive peptides and induce meat tenderization. In certain applications, the enhancement and effective control of the activity of endogenous and exogenous enzymes can be challenging but also of great technological interest.

The application of emerging technologies in meat processing could be used for the intensification of enzymatic reactions involving endogenous and exogenous enzymes. In this sense, during the last few years, there has been a growing interest in non-thermal techniques capable of accelerating enzymatic reactions without affecting the quality of meat and guaranteeing food safety [8]. Currently, some of the emerging technologies that have been used to improve the enzymatic reactions are ultrasound, pulsed electric fields (PEF), moderate electric fields (MEF), high pressure (HPP) or supercritical CO_2_ (SC-CO_2_), all of which have shown their ability to preserve the quality and safety of processed food products [9].

In this context, the aim of this paper is to review the enzymatic reactions taking place during the processing of meat products and to identify and describe those emerging technologies that have been used to intensify the enzymatic reactions in different food matrices, especially in meat products. Potential uses of these emerging technologies for enzymatic intensification in meat processing not covered in the literature will also be outlined.

## 2. Endogenous Enzyme during Meat Processing

During the processing of meat, there occurs a series of biochemical reactions, mainly catalysed by enzymes. These reactions involve enzymes responsible for post mortem glycolysis, proteolysis and lipolysis. The pH decline depends on glycogen content, and it is enzymatically controlled by enzymes such as phosphofructokinase [10]. The proteolytic enzymes involved include first muscle endopeptidases: calpains (µ-calpain and m-calpain) and cathepsins (B, H, L and D), the ubiquitin–proteasome system and, subsequently, exopeptidases such as dipeptidases, aminopeptidases and carboxypeptidases [11], which degrade the polypeptides generated by endopeptidases into peptides and free amino acids [12]. In addition to proteolytic changes, the action of lipases brings about hydrolysis reactions of triglycerides and phospholipids, which contribute to the characteristic flavour and aroma of meat [5].

### 2.1. Conversion of Muscle into Meat

Meat is the result of a series of transformations that the muscle tissue of the animal undergoes after slaughtering. This process entails structural transformations and biochemical reactions, which will produce changes affecting the technological and sensory quality of the meat. The process of converting muscle into meat comprises three stages: pre rigor mortis, rigor mortis and post rigor mortis [13]. The first stage (3–6 h) occurs immediately after the slaughter of the animal due to the interruption of blood circulation caused by bleeding. This process causes the arrival of oxygen and nutrients to be abruptly interrupted [14]. In the second stage (until 24 h), the depletion of energy components takes place, that is, adenosine triphosphate (ATP), phosphocreatinine and glucose. Finally, in the post rigor mortis, endogenous proteolytic systems lead to the disintegration of the muscle structure of the myofibrils, which induces meat tenderization. The duration of the conversion of muscle into meat depends on three aspects: the animal species, the glycogen reserves at the time the animal is slaughtered and the storage temperature. Thus, the three stages of rigor mortis take at least 14 days in cattle, 7 to 10 days in sheep, 5 to 7 days in pigs and approximately 6 h in poultry [15]. The three proteolytic systems involved in the three stages are calpains, cathepsins and ubiquitin–proteasomes [16].

In pre rigor mortis, the meat must be kept at a temperature above 10 °C until reaching the rigor mortis phase (10–10 rule, temperature within meat on a carcass should not be below 10 °C within 10 h after slaughter). Electrical stimulation immediately after the sacrifice of the animal causes a muscular contraction that accelerates the consumption of glycogen, the drop in pH and the establishment of rigor mortis. In this way, it is possible to prevent meat toughening and the apparition of the “cold shortening” problem during rigor mortis [17]. 

In pre rigor mortis, the metabolism of the animal muscle changes from aerobic to anaerobic and, therefore, undergoes a gradual decrease in energy intake. Muscle needs glycogen and phosphocreatine to synthesise ATP from glucose. Under these circumstances, the enzymes that lead the muscle metabolism begin to act, that is, those responsible for glycolysis. Among the enzymes that participate in anaerobic glycolysis, glucose 6-phosphate and phosphocreatine kinase are particularly relevant. These enzymes act until the glycogen and phosphocreatine reserves are depleted, after which ATP is reduced to form, first, adenosine diphosphate (ADP) and, subsequently, adenosine monophosphate (AMP), which can be deaminated by the enzymes responsible for the degradation of ATP. On the other hand, after the progressive reduction in ATP levels, inorganic phosphate is generated, which stimulates the degradation of glucose to pyruvate, and, subsequently, lactic acid is generated from the enzyme lactate dehydrogenase. Lactic acid causes the muscle pH to drop and the enzymes responsible for anaerobic metabolism (glycolysis) to be inactivated [18]. The decrease in muscle pH is one of the most significant post mortem changes, leading to the start of the rigor mortis. After the depletion of ATP, there is a depolarization of the membranes due to an ionic increase linked to the Ca^2+^, Na^+^ and K^+^ pumps’ standstill, which is dependent on the ATP content. That is why the Ca^2+^ ions react with troponin, which modifies the configuration of the active sites of actin; subsequently, myosin binds to actin, giving rise to the irreversible formation of actomyosin, which causes a reduction in the water retention capacity (WRC) and, therefore, a hardening of the muscle. The rigor mortis ends with the formation of actomyosin, which is characterised by muscle tension and stiffness. 

In the post rigor mortis, the enzymatic reactions responsible for the tenderization of meat take place. First, the proteolytic system of calpain plays a central role in post mortem proteolysis and softening [19]. The calpain system is dependent on Ca^2+^ and its endogenous inhibitors, calpastatins and has been described as the main factor responsible for proteolysis in the early post mortem period (0–24 h) and meat tenderization since the calpain system acts at neutral pH and its activity declines when the pH drops. Caballero et al. [20] postulated that the synergistic action of calpains with cathepsins leads to meat softening. Cathepsins, lysosomal enzymes, are activated at a lower pH than calpains; therefore, they become more important in the later phases of post mortem along with their endogenous inhibitors, cystatins. Finally, the proteasome is responsible for the degradation of most intracellular proteins, with the ubiquitin–proteasome complex responsible for the intracellular turnover of damaged proteins [21]. However, to date, the role of the proteasome in tenderization has not been fully clarified, although it is known that its activation is one of the first cellular responses to oxidative stress.

The post-slaughter evolution of pH has a great effect on the technological properties of the meat, affecting the texture (tenderization), colour and aroma due to the generation of the volatile compounds resulting from the proteolytic and lipid degradation of the meat [12]. These are reactions that will be discussed more extensively in Section 2.2 since they are of greater importance in the maturation and curing of meat. In addition, it should be noted that some factors related to genetics, nutrition and pre mortem and post mortem handling can drastically influence the conversion of muscle into meat [22].

In addition, the role played by the colour of the meat is important as it is indicative of the meat’s freshness and is, therefore, a key factor for consumer acceptance. The colour depends on the concentration and degree of oxidation of the heme compounds, mainly myoglobin (Mb). Mb is a globular protein, which can be found in four chemical forms: deoxymyoglobin (DeoxyMb), carboxymyoglobin (CarboxyMb), metmyoglobin (MetMb) and oxymyoglobin (OxyMb) [23]. Mb is a water-soluble protein, and, among the amino acid residues that it contains, histidine has received the most attention due to its key role in the structure and function of Mb. In addition, there are other heme proteins, such as haemoglobin and cytochrome C, that may also play a role in the colour of beef, lamb, pork and poultry. However, the mechanisms that control colour stability have not been completely elucidated [24]. The bright red colour of fresh meat depends on a triple balance of biochemical factors: the respiratory activities (O_2_ uptake rate), the auto-oxidation of Mb and the enzymatic reduction of MetMb, which in turn can be affected by time, temperature and muscle pH history [25]. These enzymatic processes affecting texture, colour and aroma occur in all types of meat but are particularly important in the aging of beef cuts [26]. 

Finally, the treatments carried out, both before and after the slaughter of an animal, determine the final quality of the meat and can trigger two types of meat: dark, firm and dry (DFD) and pale, soft and exudative (PSE). The factors determining this type of meat are those related to the muscle glycogen content, which affects the pH of the meat and the temperature to which the meat is subjected after slaughtering the animal [27].

### 2.2. Meat Products

There are many different meat products worldwide. The enzymatic reactions leading to meat conversion and initial meat quality influence the yield and final quality of the cooked meat products, as in the case of PSE and DFD meats [28]. However, endogenous enzymatic reactions are of less importance in cooked meat products than in raw meat products because they are inactivated by thermal treatments at temperatures above 40 °C. Accordingly, endogenous enzymes play an important role during the elaboration of dry-cured and fermented meat products. 

#### 2.2.1. Dry-Cured Meat Products

The processing of dry-cured meats typically involves the addition of curing salts, such as NaCl, and nitrates and nitrites. Nitrates and nitrites play an important role in dry-cured meat products, particularly in cured ham. The main function of these nitrifying agents is to provide food stability and safety from a microbiological point of view. In addition to ensuring food safety, they are also responsible for the formation and the stability of the characteristic colour of cured meat.

Unlike the conversion of muscle into meat, meat curing is a long process, which can be extended for up to 12 months or more in the elaboration of dry-cured ham, with enzymatic reactions being particularly relevant. As previously mentioned in Section 2.1, calpains are the enzymes that act first. These enzymes are very unstable and have an optimal pH and temperature of 5.5–6.5 and 2–6 °C, respectively [10]. Calpains are able to hydrolyse proteins, such as titin, nebulin, troponins T and I, tropomyosin and desmin [19]. On the other hand, cathepsins, along with calpains, also contribute to meat softening during post mortem, as previously mentioned. Cathepsins are mostly active at acidic pH (5.0–6.0). While cathepsins B, H and L are stable and active during the whole meat curing process, cathepsin D disappears throughout the process. To a large degree, the disappearance of cathepsin D is due to the addition of salts (NaCl) [29]. In addition, cathepsins D and L release fragments of proteins from the degradation of myofibrillary proteins, such as titin, troponins T and I and tropomyosin.

As in the case of exopeptidases, pyroglutamyl, alanyl, leucyl and arginyl aminopeptidases are enzymes that exert the greatest activity during the processing of cured meat. They present good stability during curing, although NaCl is also considered an inhibitor of these enzymes [30]. The amino acids and peptides generated during this stage by exopeptidases (glutamic acid, alanine, arginine, lysine and leucine) are responsible for the characteristic aroma and flavour of dry-cured products [31].

As in the enzymatic activity of lipases, it consists of the enzymatic hydrolysis of muscle lipids and adipose tissue to generate free fatty acids. These free fatty acids are susceptible to oxidation, which gives rise to some of the aromatic compounds typical of cured products [32]. As in the case of lipases, we can differentiate between lipases (lysosomal and neutral) and muscle phospholipases [33]. Neutral lipases act at the beginning of the curing process, forming free fatty acids. Subsequently, lysosomal acid lipase acts on triglycerides, giving rise to mono- and diglycerides and free fatty acids. Phospholipases act during the first 6 months of curing, forming free fatty acids, especially oleic, stearic, linoleic and palmitic [33].

The proteolytic activity in dry-cured meat products depends on temperature, pH and also on NaCl, which affects the proteolytic activity during the process and the final texture of the cured meat [34]. Arnau et al. [34] and García-Rey et al. [35] studied the texture of Biceps femoris salted at different contents of NaCl and observed that Biceps femoris became pastier when the NaCl content decreased. Ruiz-Ramírez et al. [36] reported that the hardness, cohesiveness and springiness of Semimembranosus and Biceps femoris muscles were affected by the NaCl content. Dry-cured muscles with less NaCl exhibited lower degrees of hardness, cohesiveness and springiness due to the fact that NaCl acts as a strong inhibitor of proteolytic activity [37].

Regarding NaNO_2_, the reaction of nitric oxide with Mb leads to the formation of nitrosylmyoglobin (NOMb), which is the pigment responsible for the reddish colouration of the dry-cured ham. The NOMb formation requires the presence of nitrites, which generate nitrogen monoxide (NO); under reducing conditions, either directly combined with Mb or indirectly in combination with MetMb, this gives rise to NOMb. This pigment is very stable, maintaining its reddish colour even in very long-lasting hams [38]. Zinc protoporphyrin (ZnPP) is a natural red pigment known for the typical colour that it imparts to the Italian dry-cured Parma ham, which is manufactured without the use of nitrifying agents. In this pigment, the iron ion of the porphyrin ring has been replaced by a zinc ion. There is evidence that, in dry-cured hams, ZnPP is mainly formed endogenously due to the enzyme ferrochelatase (FeCH) [39]. The mechanisms of the formation of ZnPP in Parma hams have recently been reviewed [40]. However, it has been shown that ZnPP can also be formed in different quantities in Iberian and Serrano hams during their processing [41,42,43]. In this regard, the possible relationship between ZnPP and lipolysis or proteolysis has been investigated in hams and other meat models [43,44,45]

#### 2.2.2. Fermented Sausages

There is a wide variety of dry-cured products without anatomical integrity, with or without fermentation. Products that present a fermentation stage during their processing undergo additional enzymatic reactions that will be outlined in this section [46]. The final characteristics and quality of these fermented products depend on the raw material, the microbial population as well as on the processing conditions during fermentation (temperature, 18–26 °C; relative humidity, 90–95% and time, 24–72 h). Microorganisms involved in fermentation include the microbiota of the raw meat and microorganisms added as starter cultures (lactic acid bacteria (*Lactobacillus*), Gram-positive catalase-positive cocci (*Staphylococcus*), yeasts and moulds). Lactic acid bacteria, essentially *Lactobacillus sakei*, play an important role in the technological properties and microbial stability of the final product through the production of lactic and acetic acids and the consequent decrease in pH to approximately 5. At this pH, muscle proteins coagulate and lose their water-holding capacity, leading to an increase in the firmness and cohesiveness of the final product. In addition, the accumulation of lactic and acetic acids inhibits the growth of pathogenic and spoilage microorganisms. On the other hand, Staphylococcus also plays an important role in the fermentation process since it contributes to the development of the characteristic flavour and colour together with the acidic pH promoted by the lactic acid bacteria, which improves the colour stability of the fermented products. The action of these microorganisms (lactic acid bacteria and *Staphylococcus*) is based on the endopeptidase and exopeptidase enzymes that they generate. Overall, these endopeptidases and exopeptidases contribute to an increase in the concentration of free amino acids that affect flavour development [47]. Finally, yeasts and moulds participate in fermentation through lactate oxidation and the enzymatic reactions of proteolysis and lipolysis [48].

During sausage fermentation, muscle proteins (actin and myosin) begin to degrade the peptides, mainly through cathepsin D, while, at the same time, lipolysis begins. Both the microorganisms (lactic acid bacteria and Staphylococcus) added as starter cultures and the meat endogenous enzymes (lysosomal lipases and phospholipases, explained in Section 2.2) produce lipolysis, which generates free fatty acids; due to successive modifications, these give rise to esters, aldehydes and ketones, among other compounds, and participate in the final aroma of the fermented product [49].

Once the fermentation stage of sausages is complete, the maturation stage begins. This stage implies the maintenance of the sausages during variable periods under controlled relative humidity and temperature conditions. The most common procedures usually consist of 5–10 days at 18–22 °C and a relative humidity of 80–90%; subsequently, they are kept at 12–15 °C and a relative humidity of 65–80%. The maturation stage can range from 20 to 90 days depending on the type of sausage [31]. During maturation, the proteolysis initiated in the fermentation stage continues through the action of exopeptidases, both of endogenous and microbial origins, which release peptides and free amino acids [50]. In addition, the lipolysis initiated in the fermentation continues. Subsequently, oxidative processes involving the release of free fatty acids and the oxidation of unsaturated fatty acids, particularly polyunsaturated acids, along with the production of carbonyl compounds, take place [46].

## 3. Exogenous Enzymes in Meat Processing: The Addition of Exogenous Enzymes in Meat Processing

### 3.1. Restructured Meat Products

Meat industries are currently processing innovative products, such as restructured meat. By means of restructured meat, an attempt is undertaken to mimic the appearance of meat muscle. Thereby, restructured meat is considered an intermediate product between minced meat and a piece of meat with anatomical integrity. Its production begins with meat pieces of different sizes to achieve a consistent product by joining these pieces [51]. Using this process, it is possible to produce products of wider consumer acceptance from portions of meat of low commercial value, with poor texture and difficult commercialization. The restructuration technology is applied to all types of meat.

Different blinders are used in the manufacturing of restructured meat products. One of the most important ingredients is the enzyme transglutaminase [52]. Transglutaminase is an enzyme typically obtained from the microorganism *Streptoverticilium mobaraense* despite being naturally found in most tissues of living organisms. This enzyme has a range of action between 0 and 60 °C, the optimal being at 50 °C and pH 7. In addition, unlike endopeptidase enzymes such as calpain, it is independent of Ca^2+^ and has the ability to improve the functional characteristics of protein, including water retention and water solubility, and the functional properties, such as food taste and toughness [53,54]. Transglutaminase is a transferase enzyme that is characterised by catalysing cross-breeding reactions between the residual γ-carboxyamide residual groups of glutamine and the residual ε-amino groups of lysine, resulting in a molecular cross of proteins by forming ε–γ glutamyl lysine bonds [55]. The inter- and intramolecular bonds they form are highly resistant to proteolysis, which is why transglutaminase becomes an important ingredient that enhances the physical and functional properties of meat products [56]. Transglutaminase has been tested on a large number of meat products with different objectives, one of the which is to develop new meat products or enriched meat products. Thus, Baugreet et al. [57] optimised a restructured beef fillet enriched with vegetable protein (lentil and rice) through the action of transglutaminase, from which meat with a high protein value (28 g protein/100 g meat) was obtained. Ahhmed et al. [58], investigated the improvement in the physical properties of meat products, such as the texture of chicken and beef sausages. In addition, there are several studies that show how to achieve a good bond between meat pieces using transglutaminase, without the need for the use of salt or phosphates, for the purposes of manufacturing cooked ham [59] and sausages [60]. This enzyme has also been used in restructured unsalted and low-fat kebab meat [61], improving its functional properties.

### 3.2. Bioactive Peptides

It is known that bioactive peptides are generated through the enzymatic hydrolysis of whole protein molecules. The activity of bioactive peptides derived from meat proteins depends on the amino acid sequence and can affect the cardiovascular, immune, nervous and digestive systems [62]. Peptides with antihypertensive activity act by inhibiting the angiotensin I converting enzyme. The muscle proteins of pork (myosin, actin, troponin T and titin) are a source of antihypertensive peptides, which can be generated by the action of gastrointestinal enzymes (pepsin and pancreatin) [63,64]. Kim et al. [65] studied the enzymatic activity of five proteases (alcalase, chymotrypsin, neutral, pronase E and trypsin) on bovine gelatine and found that this protein source contributed to the formation of bioactive peptides, inhibitors of angiotensin I converting enzyme. Likewise, Arrutia et al. [66] studied serum albumin, the main blood protein. The hydrolysis of this protein by trypsin resulted in bioactive peptides that inhibited angiotensin I converting enzyme (antihypertensive activity) and DPP-IV (glucose regulation) and exhibited antioxidant activity. 

In addition to the generation of peptides by the digestive enzymes from meat, hydrolysates can be obtained by using enzymes, such as papain, bromelain, thermolysine, actinase E or proteinase K [11]. The peptides resulting from the enzymatic action of papain and actinase E on the myofibrillary proteins of pork exhibit antioxidant activity in a peroxidation system with linolenic acid, induced by Fe^2+^ [67]. Li et al. [68] and Wang et al. [69] purified and characterised bioactive peptides with antioxidant activity from duck by adding a neutralase and in mutton from endogenous enzymes, respectively. Kim et al. [70] compared the antioxidant activity of various peptides resulting from the addition of six enzymes (papain, pepsin, trypsin, chemotrypsin, alcalase and neutral) to venison meat, the papain hydrolysates being those exhibiting the greatest antioxidant activity. Therefore, it may be possible to purify and design food ingredients through the addition of enzymes.

### 3.3. Tenderization

The tenderization process is the result of the proteolysis of muscle proteins by three large study systems, which are cathepsins, calpains or calcium-dependent peptidases and proteasomes (discussed in Section 2) after rigor mortis. On the other hand, meat aging is a process during which exogenous enzymes and microorganisms act upon the meat to break down the connective tissue, thereby tenderizing the meat and giving it a richer flavour [71]. Tenderness has been rated by consumers as the most important organoleptic attribute of fresh meat. Regarding exogenous enzyme systems, they are mostly formed by enzymes of plant origin, papain, bromelain and ficin being the most widely studied. Other less commonly studied enzymes are zingibain, cucumisin and actinidin [72]. Other authors have reviewed the source, mode of action and optimal working temperatures of these exogenous enzymes [73].

Papain can be found in the latex of the papaya plant, Carica papaya, and is an enzyme that protects the papaya plant from insects [74]. Papain is a very heat-stable enzyme, and, therefore, it is not easily deactivated, allowing a continuous change in product texture even after cooking [75]. The application of papain for beef tenderization purposes has been studied at different concentrations (0.003, 0.005, 0.007 and 0.01 mg/100 g meat), allowing the enzyme to act from 24 to 48 h at 4 °C with a subsequent heat treatment at 83 °C for 10 min. The results showed an increase in tenderness and juiciness as the concentration rose and the application time lengthened. Ashie et al. [76] showed how, in beef, the application of papain to obtain a residual level of 0.002 to 0.05 units of proteolytic activity/100 g meat improved the tenderness of the meat by 25–30%. However, high doses of papain are not recommended because they can lead to meat pastiness [77]. 

Bromelain is a complex of proteolytic enzymes found in some fruits, specifically in the stem of pineapple (*Ananas comosus*), whose enzymatic activity is slightly more limited than that of papain. The profile of proteins subjected to enzymatic action indicates that papain degrades myosin and actin at similar rates, while bromelain mainly degrades myosin [78]. Ionescu et al. [79] studied the effect of applying different concentrations of papain and bromelain (10, 15 and 20 mg enzyme/100 g of meat) to beef for 24 and 48 h at 4 °C; it is subsequently cooked, which leads to an improvement in the meat softening process that is more noticeable when it is papain that is applied rather than bromelain. The optimal concentration applied was 10 mg enzyme (papain or bromelain)/100 g meat for a tenderization time of 24 h at 4 °C in order to avoid an excessive structural degradation of the meat caused by the enzymatic treatment.

Ficin is a proteolytic substance obtained from the latex of trees of the genus Ficus. The cysteine proteases of Ficus glabrata and Ficus carica have demonstrated an ability to increase the solubilization of proteins and to improve the tenderness of meat products. Ramezani et al. [80] observed how the quality of beef sausages could be improved by using (the enzyme) ficin together with chemically modified soy proteins. It was also observed how (the enzyme) ficin increased the solubility of meat proteins by degrading them into units of smaller molecular weight. In addition, the joint action of ficin and soy proteins boosted the softening effect of the meat compared to what may be achieved through their individual effects. This observation suggests that the addition of enzymes can be combined with other ingredients and processes to enhance the properties of the final products.

Zingibain is a vegetable proteolytic enzyme isolated from the ginger rhizome. The ginger rhizome is mainly used as a flavouring agent, but its application as a softening agent in meat products is of great interest. In this regard, although zingibain showed good potential for beef softening, the injection level was limited due to problems associated with taste [81]. Consequently, if the enzyme could be purified, its application in meat would be more promising [82].

On the other hand, cucumisin is a proteolytic enzyme of Cucumis trigonus Roxb (Kachri) plants that grow wild in India, Afghanistan and Persia [83]. Naveena et al. [84] studied the application of the enzymes cucumisin, zingibain and papain in buffalo meat. The results obtained showed the softening effect of the three enzymes and that the samples treated with zingibain were rated as superior, attributed to a desired ginger taste. Regarding the samples treated with cucumisin and papain, they were rated equally. Therefore, cucumisin and zingibain, which are cheaper enzymes, could be used as an alternative to the use of papain.

The enzyme actinidin is obtained from kiwi juice. Actinidin hydrolyses microfibrillar proteins, leading to new peptides and the activation of m-calpain during the post mortem aging period [85]. The effect of raw extracts and purified actinidin of the Xuxiang cultivar was studied at a concentration of 0.25 and 0.5 mg/100 g meat in pork and rabbit Longissimus dorsi. After the injection of the enzyme, a period of action (3 h at 20 °C) and cooking (75 °C for 30 min), the samples treated with actinidin showed a higher shear force in the texture tests than those treated with papain, especially in the pork samples, with lower shear forces compared to the control sample without the addition of exogenous enzymes due to higher myosin degradation [86]. Moreover, Christensen et al. [87] showed that an actinidin injection accelerated the muscle-to-meat conversion process, improving the tenderness in the Biceps femoris muscle of the pork, affecting the protein myofibrils and the proteins of the connective tissue without affecting the flavour and juiciness of the meat. 

In addition to exogenous enzymes of plant origin, there are also microbial enzymes, such as *Bacillus subtilis* elastase and *Aspergillus oryzae* enzymes, that can be used for meat softening. Qihe et al. [88] compared the action of *Bacillus spp* elastase with that of papain proteases, observing a significant degradation of meat myofibrils, which indicates that it could be used as a substitute of papain. As for the applications of the microorganism *Aspergillus oryzae*, Ashie et al. [76] studied the enzyme aspartic protease (AP) expressed in *Aspergillus oryzae*, the use of which was shown to be of interest in the beef softening process compared to papain. The softening effect of AP occurs mainly during cooking and not in the cold storage, as in the case of papain. That is why the injection of this enzyme could be of interest in the storage of packaged fresh meat products. Sullivan and Calkins [82] conducted a study into the degree of softening of the muscles, Triceps brachii and supraspinous, using seven types of enzymes (papain, ficin, bromelain, zingibain, *Bacillus subtilis* protease and two proteases from *Aspergillus oryzae*: *Aspergillus oryzae* concentrate protease (ACONC) and *Aspergillus oryzae* 400 protease (A400). The results showed a high degree of meat softening for all the enzymes. Regarding the mode of action of the proteases, those of vegetable origin led to a balanced degradation of degraded myofibrillary and collagen proteins; those of microbial origin, meanwhile, tended to degrade more myofibrillar proteins compared to collagen and in turn provided better sensory results than those of vegetable origin.

## 4. The Intensification of Enzymatic Reactions through the Use of Emerging Technologies

Given the growing demand for products that guarantee food safety and the quality of the final product, as well as the interest of the meat industry in process intensification, new technologies are emerging. These technologies aim to improve existing processes in terms of process rate, energy consumption or final product quality. The use of novel technologies may also pursue the development of new processes to bridge the gaps left by those presently in use. In this section, the use of emerging technologies that focus on the intensification of enzymatic reactions, especially in meat products, will be addressed.

### 4.1. Ultrasound

Ultrasound is an emerging technology that has applications both in the analysis and in the intensification of food processes. Ultrasound is an elastic wave with frequencies higher than the human ear detection limit (20 kHz). Power ultrasound (US) applications work in the frequency range of 20 to 100 kHz. When US waves are transmitted in liquid media and reach a power threshold, they induce the transient growth of air bubbles and their collapse within liquids, a phenomenon known as cavitation. Another phenomenon resulting from the bubble size variation and subsequent collapse is the development of strong micro-streaming currents, associated with high-velocity gradients and shear stresses that alter the media properties and can reduce the external resistance to mass transfer by increasing the bulk transport within the fluid [89]. In addition, US cavitation can break down water molecules, generating highly reactive free radicals that may react with and modify other molecules [90]. Therefore, the effects produced by US may induce physical and chemical effects, which can be used in the food industry to intensify extraction processes, heating/cooling processes, microbial inactivation, drying and chemical and enzymatic reactions, among other applications. 

In the field of enzymatic reactions, US was applied to different food products (Table 1). Şener et al. [91] reported that ultrasonic treatment in β-galactosidase from *Kluyveromyces marzianus* achieved a lactose hydrolysis in milk of 90% compared to 84% without sonication. In addition, a residual enzymatic activity of 75% was obtained (only 25% of the enzyme activity was lost) under optimal operating conditions (37 °C, pH 6.7, 20 W and 20 kHz) after 30 min of sonication. In addition, in the field of sugar hydrolysis, the application of US (25 Hz, 22 W/L, 40 °C) in sucrose solutions enhanced the reaction rate of the carbohydrate hydrolysis with invertase by 33.0% compared to the treatment without US [92]. On the other hand, Wang et al. [93] reported that the application of US together with glucoamylase accelerated the degree of starch hydrolysis, decreasing the molecular weight of starch by 80.19% and increasing solubility by 136.5%. The optimal conditions were 35 °C, 40 min, an ultrasonic intensity of 7.2 W/mL and a frequency of 22 kHz. Another improvement brought about by the application of US in the hydrolysis of carbohydrates was in the hydrolysis of pectin. The application of US, together with pectinase from *Aspergillus niger*, improved the reaction rate of the enzymatic hydrolysis process of citrus peel pectin to obtain galacturonic acid, increasing the rate of hydrolysis by 32.59% over the control induced by enzyme conformational changes that resulted in an increased affinity for pectin. The process was carried out by applying an intensity of 4.5 W/mL and a frequency of 22 kHz for 10 min at 20 °C [94]. In addition to an intensification in the activity of the enzymes that hydrolyse carbohydrates, ultrasound has also been used to accelerate protein hydrolysis [95]. In this latter study, the degree of protein hydrolysis and enzyme recovery in soy sauce went from 10.46% and 57%, respectively, without US treatment (20 min, 50 °C) to 15.44% and 61.94%, respectively, with US treatment (126.4 W/cm^2^, 20 min, 50 °C). When the treatment time was extended to 240 min, US increased the degree of hydrolysis by an additional 3.5% and the recovery by 4.79%, compared to the 20 min treatment. In these applications, the great energy release caused by the acoustic waves helped to improve both hydrolysis and the enzyme extraction from the inner cell [96,97]. However, the use of high power may also cause enzyme denaturation, and, thus, another alternative strategy is the application of low or moderate US treatments in order to induce a mild cavitation, or only a micro-stirring, and promote the union of the substrates with the active sites, or even product diffusion without altering the enzyme structure [98].

US has also been used to improve different meat properties, promoting safer and better-quality meat products [99]. Specifically, US has been used to improve the texture of raw and processed meat (Table 1) by contributing to the release of myofibrillar proteins, which play a fundamental role in the formation of gels and are responsible for the water retention capacity, the emulsifying properties and the tenderness of meat [100]. US cavitation is also capable of breaking down cell components, causing the softening of cell membranes. The alteration of the meat tissue results in the extraction of proteins and other compounds outside the cells and the consequent acceleration in the enzymatic activity [101]. Got et al. [102] reported that, when US was applied (two consecutive periods of 15 s allowing a rest period of 2 min; 2.6 MHz; 10 W/cm^2^) to beef Semimembranosus muscles in pre rigor mortis, the treatment accelerated the release of the lysosomal enzyme due to membrane fragmentation, as well as β-glucuronidase, and weakened the muscle structure. In addition, Xiong et al. [103] observed a significant tenderisation of the hen breast muscle following the application of US (24 Hz, 12 W/cm^2^, 15 s) and demonstrated that the effect was linked to muscle degradation by the combination of US and proteases (calpain and cathepsin). Lima et al. [104] studied the combined use of US (135 W, 40 kHz, 10 min) and the papain enzyme in chicken breast, observing a reduction in hardness, leading to an improvement in tenderness. In the same vein, Mehrabani et al. [105] observed an increase in proteolytic activity in beef, improving tenderness when combining US (100 W, 37 kHz, 20 min) and leek extract proteases.

In addition to the improvement in meat texture due to the combined action of ultrasound and enzymes, US has also been used in meat products to accelerate freezing [106] and thawing [107] or drying by means of mild thermal treatments to correct the defect of pastiness in dry-cured ham [108]. Finally, US has also been applied for the purposes of accelerating the enzyme extraction, such as in the case of the enzyme ferrochelatase from pork liver [109], or even the penetration of enzymes into meat. In this regard, Barekat and Soltanizadeh [110] reported that the use of ultrasound (20 kHz; at 100 W for 20 min) to treat *Longissimus lumborum* of beef, immersed in a 0.1% papain solution, improved the enzyme diffusion in the deepest layer of the meat by 62% compared to untreated meat. Finally, US enhanced the diffusion of salt and water in pork *Longissimus dorsi* [111,112]. Therefore, this improved salt diffusion could also help to accelerate (boost) the action of proteases in the elaboration of dry-cured meat products. 

### 4.2. High Pressure

High-pressure processing (HPP) is a technique that is based on the application of an elevated and uniform high hydrostatic pressure (300–700 MPa) for a short time (from a few seconds to several minutes) to a food product by means of a transmitting liquid (typically water) (added two more citations to clarify these issues [113,114]). By using HPP, food can be processed at room temperature or even cooler. This is a significant advantage, which is common to other non-thermal technologies, since it allows the sensory and nutritional properties of the treated food to be maintained [115].

Mostly, high pressure causes membrane proteins denaturation and membrane disruption, all causing changes in the permeability of cell membranes, as well as the denaturation of proteins and the inactivation of some degradation enzymes and vegetative microorganisms, reasons why it has been mainly used in food preservation processes [116]. However, the enhancement of enzymatic reactions in food products using this emerging technology has also been studied (Table 2). Some of the enzymatic reactions that HPP enhances are those related to the enzymatic hydrolysis of proteins in dairy products. Saldo et al. [117] reported the acceleration in the maturation of goat cheese, in particular, the process of proteolysis by which casein, the main protein in milk, is hydrolysed into free peptides and amino acids due to an enhanced enzyme activity. The maturation was completed in 14 days with HPP, while typical maturation took 28 days. The optimal conditions studied were 400 MPa for 5 min. Hayashi et al. [118] studied the hydrolysis of the β-lactoglobulin by the thermolysin enzyme, observing that this enzyme only partially hydrolysed the β-lactoglobulin in cow’s whey concentrate at atmospheric pressure and 25 °C. However, after the HPP treatment at 200 MPa for 180 min, the β-lactoglobulin was completely hydrolysed, likely due to protein’s susceptibility to denaturation. In addition to acting on whey proteins, Kim et al. [119] reported that the application of a pre-treatment with HPP (50 MPa for 240 min at 55 °C) to a mixture of a peptinase cocktail and black garlic juice, before its aging process, increased the enzymatic activity of peptinase to 5 units/mL compared to 3 units/mL without HPP. Furthermore, the concentration of galacturonic acid released was higher (4.5 mM) than in the untreated black garlic juice (2 mM). The application of the pre-treatment permitted an improvement in the extractability and clarification of the black garlic juice. In addition, it enhanced the functional properties of black garlic with the increase in S-allylcysteine with antidiabetic activity. 

Regarding the intensification of enzymatic reactions that take place in meat as a result of using HPP (Table 2), Jung et al. [120] reported the effect of high pressure on the colour of the beef Biceps femoris muscle. These authors showed that MetMb can be reduced at moderate pressures (130 MPa) and low temperatures (10 °C) through the activation of the enzymatic system involved in the reduction of MetMb, whereas, at higher pressures, the enzymatic system is disturbed. The HPP enhanced this enzymatic reaction and consequently improved the redness and the typical colour of fresh meat. Furthermore, Rivas-Cañedo et al. [121] studied the influence of HPP (600 MPa for 6 min) on lipid oxidation, aminopeptidase and free amino acid activities in dry-cured ham of differing chemical compositions after 5 months at 4 °C. HPP increased lipid peroxidation rates and reduced the activity of all the aminopeptidases studied. The enzymes may have been denatured by the relatively intense pressurization treatment. Finally, it is worth mentioning the review of Roobab et al. [114] on the effect of HPP on the muscle of fish and shellfish; in this case, HPP has been shown to have potential industrial applications related to freezing and thawing processes, hardness and others.

In addition to the enhancement of enzymatic reactions, it is worth highlighting the application of HPP in improving tenderness, such as a means of improving tenderness. The effects of HPP on meat tenderness have been addressed by other authors [122,123,124]. Sikes et al. [125] and Ma and Ledward [126] showed that the HPP treatment (200 MPa, 20 min and 60 °C) modified the structure of the myofibrils of *Sternomandibularis* and *Longissimus dorsi* muscles of beef and led to changes in tenderness. It seems that pressures above 200 MPa cause the lysosomes to rupture, leading to higher cathepsins concentrations in the cytoplasm, which are quite stable to pressure treatments. On the contrary, Jung et al. [127] demonstrated that pressures between 100 and 600 MPa at low temperatures (10 °C) modified the structure of the myofibrils of the Biceps femoris muscle of beef without producing changes in tenderness. However, the same authors reported that the activity of cathepsin D and acid phosphatase in pressurised meat samples was higher than in controls throughout storage [127]. Thus, the HPP effect on meat tenderness seems to be pressure-dependent to allow the release of the enzyme and thereafter time–temperature-dependent to allow the activity of proteases.

### 4.3. Electrical Stimulation

In recent years, the use of electric fields for food processing has been receiving special attention [128]. Pulsed (PEF) and moderate electric fields (MEF) constitute the two main modes of electrical stimulation applied in the food industry.

#### 4.3.1. Pulsed Electric Fields (PEF)

This emerging non-thermal technology is based on the application of electrical energy through high-intensity pulses of short duration (µs or ms) and high voltage (0.1–40 kV/cm) [129]. By applying a high-energy electric field, the reversible or irreversible permeabilization of cell membranes can be achieved, a phenomenon known as electroporation [130].

The electroporation of animal or plant tissue membranes has numerous applications in those processes of the food industry in which mass transfer takes place through cell membranes, such as solid–liquid extraction, pressure extraction, dehydration, osmotic dehydration or the curing and marinating of meat and fish [131]. However, PEF may also affect the enzymatic activity of several reactions (Table 3). Electrochemical reactions might occur on the surface of the electrode, causing electrolysis and, therefore, a change in pH, which leads to enzyme inactivation or a slowdown in enzymatic activity [132]. For example, papain activity was reduced by the electric field strength (20–50 kV/cm and pulses 200–500) due to the oxidation of cysteine amino acid residue located in the active site of papain [133]. The activity of ascorbic acid oxidase in carrots was reduced up to 61% after the application of pulsed electrical energy of 516 kJ/kg at electric field strength of 0.6 kV/cm [134]. Similar reductions were observed in pectin methylesterase activity in tomatoes [135] and in polyphenoloxidase from apples and pears [136].

PEF has been used in the meat industry to improve meat tenderness (Table 3). In venison, calpain was observed to undergo marked proteolysis in two different treatments (0.2 kV/cm, 1.93 kJ/kg, 20 μs and 0.5 kV/cm, 70.2 kJ/kg and 20 μs) [137]. The application of high-electric-field-strength PEF treatments (10 kV, 90 Hz, 20 μs) in beef Semimembranosus improved tenderness after a 3-day post mortem maturation period [138]. This is due to the phenomenon of electroporation, which allows Ca^2+^ release, which activates its dependent proteases, calpains [124,139]. However, the application of PEF at lower electric field intensity (0.2–0.6 kV/cm, 1–50 Hz, 20 μs) in the Longissimus thoracis muscle of beef brought about changes in the microstructure of the meat, but these changes did not affect the activity of proteases, and, therefore, meat tenderness was not modified after 1–3 d post mortem [140]. On the other hand, Arroyo et al. [141] showed that the application of PEF (3 kV/cm, 300 pulses of 20 μs) on chicken meat did not improve its tenderness post mortem (1 day). Thus, applying PEF for the purposes of improving the meat tenderness depends on both the electric field strength and the state of the post mortem maturation period.

In addition to enhancing the enzymatic reactions to improve the texture of the meat, PEF treatments have been used to reduce the thermal stability of intramuscular collagen due to the physical alteration in the muscle fibres and thus to improve the tenderness of the meat [142]. On the other hand, treatments of PEF (1.4 kV/cm, 90 Hz, 20 μs) before freezing–thawing improved the volatile profile and sensory properties of cooked lamb meat [143]. Kantono et al. [144] showed that PEF application (1–1.4 kV/cm; 88–109 kJ/k, 90 Hz, 20 μs) to frozen thawed lamb meat after 7 days refrigerated storage produced an increase in the amino acid content, which can be attributed to the release of lysosomal cathepsins and reduction in the fatty acid content of these cuts of lamb, which was related to the progression of lipid oxidation. Therefore, the use of emerging technologies, under controlled conditions, could allow an acceleration in the enzymatic processes alone or in combination with other processes and, therefore, an increase in the yield and quality of the meat products. 

#### 4.3.2. Moderate Electric Fields (MEF)

Moderate electric fields (MEF) are based on the application of electrical energy in a range of 1 to 1000 V/cm using an alternating current with arbitrary frequencies (50–25,000 Hz) and waveforms [98]. Unlike the application of PEF, it can have ohmic heating effects, that is, a thermal process where the passage of a moderate electric current through the food causes heating [145]. Through the application of MEF, although with a lower intensity than for PEF, cell electroporation can also take place [146].

The ohmic heating and the electroporation of cell membranes have allowed the use of MEF as a means of intensifying extraction [147]. In addition, it has also been reported that low-intensity MEF (up to 12 V/cm, 50 Hz) increases enzymatic activity by improving the molecular mobility associated with temperature (ohmic heating), thus improving mass transfer within the system [148]. In this sense, the application of MEF in non-meat products improved the enzymatic reactions (Table 4). In the hydrolysis of corn starch (0–10 V/cm, 50 Hz, 30 min, temperature was not controlled), MEF caused an increase in hydrolysis, activating glucoamylase [149]. In addition, it was observed that the application of MEF (5.8 V/cm, 60 Hz sinusoidal wave, reaching 80 °C starting at 65 °C) in tomato homogenate activated pectin methyl esterase (PME) [150]. However, the application of MEF can lead to high temperatures that might cause enzymatic inactivation due to conformational changes in the enzyme structure [151]. In the case of sugarcane juice treated with MEF (98 °C, 3.57 V/cm, for 12 min), peroxidase and polyphenol oxidase were completely inactivated [152], while, in pea puree, the application of MEF (50 V/cm, for 54 s up to 100 °C) involved the inactivation of the peroxidase enzyme [153].

In the meat industry, MEF has also been used to intensify the tenderization of meat by accelerating the ageing process (Table 4). The stability of the enzymes should be addressed in future studies. It has been reported that the electroporation caused by MEF can accelerate the post mortem glycolysis process in which the production of lactic acid takes place, ensuring that the pH of the meat drops below 6.0, before the muscle temperature reaches 10 °C [154]. In bovine Sternomandibularis, an improvement in tenderness and the prevention of carcass hardening by accelerated post mortem glycolysis was demonstrated after the application of moderate electrical stimulation (10–600 V, 50 Hz, 2–100 pulses/s, pulse shapes: sinusoidal or square wave, polarities: alternating or unidirectional and stimulating periods of 5–120 s) and a subsequent aging period [155]. Moreover, it has been shown how the application of MEF caused the electroporation of the muscle cell membrane, affecting the release of Ca^2+^ and the activation of calpain proteases in the post mortem maturation period. In beef, this phenomenon was observed by applying 550 V, 60 Hz, 1 s pulse and 0.5 s rest for 120 s [156].

In addition to enhancing the enzymatic reactions in order to improve the meat texture, MEF has also been applied for the purposes of improving the diffusion of NaCl in porcine muscle [157] and the quality of the pork carcass during cold storage in modified atmosphere packaging [158], and for cooking meat and meat products using ohmic heating [159]. 

### 4.4. Supercritical Fluids

This methodology is based on the use of a fluid, normally carbon dioxide, at temperatures and pressures above its critical point (Pc = 73.8 bar and Tc = 31.1 °C, for CO_2_). The most widespread application of supercritical fluids in the food industry is the extraction of target compounds from vegetal or animal matrices. 

Applications involving supercritical fluids and enzymes in food-related processes include the improvement in the enzymatic activity of enzymes in supercritical CO_2_ (Table 5). In this regard, Lee et al. [160] investigated the reaction of enzymatic hydrolysis with supercritical carbon dioxide (SC-CO_2_) as a reaction medium in order to produce glucose from starch. The rate of enzymatic reaction improved with the application of SC-CO_2_, with the optimal process conditions being close to the critical point of SC-CO_2_ (31 °C, 73.8 bar), which can be attributed to an enhanced solvation state and mass transfer. Hojnik Podrepšek et al. [161] also experimented with immobilised cellulase and analysed the effects of SC-CO_2_ on the free and immobilised enzyme and a control treatment without using SC-CO_2_ at different combinations of pressure (atmospheric, 10 and 20 MPa), temperature (40 and 50 °C) and different reaction times (1, 3, 4 and 24 h). The maximum enzymatic activity was reached with the immobilised enzyme at 50 °C and 10 MPa, which increased the enzyme activity by 57% after 3 h treatment compared to a treatment without SC-CO_2_. All the reactions in SC-CO_2_ (with the free and immobilised enzyme) exhibited greater enzymatic activity than those carried out in a non-supercritical medium. The better performance in the supercritical medium could be attributed to an increase in enzyme activity and stability due to changes in the conformational structure of the enzyme. On the other hand, Senyay-Oncel and Yesil-Celiktas [162] evaluated the effect of supercritical SC-CO_2_ as a pre-treatment on the enzymatic activity of α-amylase. The optimal process conditions were 240 bar, 41 °C, 4 g/min CO_2_ flow and 150 min of treatment time, providing 67.7% higher enzyme activity (29.728 μmol/mL·min) when tested on a starch solution than in the enzyme not treated with CO_2_ (17.726 μmol/mL·min). Thus, the use of SC-CO_2_ as a pre-treatment of the α-amylase also improved the enzymatic activity when applied in food samples.

Conversely, there is one study in the literature reporting the inactivation of various enzymes as a result of SC-CO_2_ [163]. To the best of our knowledge, there are no studies focusing on the effect of SC-CO_2_ on meat enzymes; however, supercritical fluids have been applied for the extraction and detection of pesticides by enzyme immunoassays [164,165]. In addition, SC-CO_2_ has been used for the lipid extraction in pork and lamb meat [166]. Moreover, the extraction of traces of antibiotics in meat [167] in a supercritical medium has also been reported. SC-CO_2_ can also be used in combination with US and saline solution for the microbial inactivation of *Escherichia coli* in dry-cured ham, with no significant changes in colour, texture or pH [168]. Morbiato et al. [169] also combined US and SC-CO_2_ technologies to jointly dry chicken breast, preserving nutritional quality and inactivating mesophilic bacteria, moulds, yeasts and *Salmonella* spp.

## 5. Conclusions and Future Trends in the Intensification of Enzymatic Reactions in Meat Processing

Emerging technologies enhance enzymatic reactions through different mechanisms. Specifically, the cycles of compression and expansion and the consequent cavitation caused by ultrasound (US) may accelerate mass transfer and cause the rupture of cell walls, thereby improving the binding of the enzyme with the substrate and consequently enhancing the rate of enzymatic reaction. In addition, the micro-agitation caused by moderate-intensity US (without reaching cavitation that could result in enzyme inactivation) leads to an improvement in the diffusion of the products resulting from the enzymatic reaction, as well as to the diffusion and improved interaction between the enzyme and the substrate. High-pressure processing (HPP) may cause changes in the internal structure of the matrix and cell disruption, both of which can enhance the binding of the enzyme with the substrate. Likewise, by the application of electrical stimulation, electroporation may cause the rupture of cell membranes, which leads to a release of enzymes and substrates that facilitate their subsequent bonding. Finally, supercritical fluids, particularly supercritical CO_2_ (SC-CO_2_), can be used to promote greater enzyme activity and stability due to changes in the conformational structure of the enzyme. The performance of the different technologies in meat processing should, however, be examined for particular potential applications.

Emerging technologies can be used to shorten and intensify the enzymatic reactions that take place during meat processing. Between the slaughter of the animal and rigor mortis, the first enzymatic reaction that occurs is glycolysis, a stage in which the energy reserves are depleted and the pH drops. To date, it has been shown that the application of MEF accelerates the formation of lactic acid from glycogen. In addition, moderate-intensity US could be applied in order to reduce the activation energy of the enzymes involved in the glycolysis reactions and thus promote the binding between the enzyme and the substrates; this accelerates the rate of reaction and diffusion of lactic acid with the consequent drop in the muscle pH. However, other emerging technologies, such as SC-CO_2_, where temperatures must be above 31 °C, would not be feasible due to the potential negative temperature effect of their application. 

Tenderness is an important factor, one that determines the quality of the meat. US, HPP as well as electrical stimulation by PEF and MEF have been applied to intensify the enzyme reactions related to this attribute. As regards US, its application causes the alteration of the cell membrane, leading to the release of more myofibrillary proteins outside this membrane and its consequent enzymatic acceleration by calpain and cathepsin. HPP has also been used to modify the structure of muscle myofibrils by enhancing its contact with enzymes. Finally, the application of PEF and MEF, through the electroporation of the cell membranes, enhances the release of Ca^2+^ and activates the calpain Ca^2+^-dependent enzymes. The SC-CO_2_ has not been applied so far; however, its application could improve the proteolysis of endopeptidases by causing changes in the cell membrane structure, enhancing the release of proteins into the medium. 

As far as the colour of raw meat is concerned, HPP has been applied to intensify the enzymatic reaction of MetMb reductase, which catalyses the reduction of MetHb back to OxyMb, thus enhancing meat redness. Another technology that could intensify the enzymatic reaction of MetMb reductase would be US at moderate intensities since it could limit the temperature increase and the micro-agitation generated by US could reduce the activation energy, intensifying the binding of MetMb reductase with Mb. In addition, as regards the colour of dry-cured products, it has been shown that the formation of zinc protoporphyrin (ZnPP) in Parma ham takes place from the reaction catalysed by the enzyme FeCH. This enzymatic reaction takes place during the long curing process, up to 2 years, which entails drying and further aging. However, the application of HPP and moderate-intensity US could accelerate the enzymatic reaction following similar mechanisms to those in raw meat. In addition, US has been used to enhance the extraction of the enzyme FeCH from pork liver and to intensify the formation of ZnPP from pork liver and blood, which is considered to be a potential colouring ingredient for the meat industry. This technology could also be used to accelerate the formation of ZnPP in dry-cured meat products.

In the meat curing and aging process, other proteolysis reactions catalysed by exopeptidases (dipeptidases, aminopeptidases and carboxypeptidases) and lipolysis (lipase and phospholipases) take place, giving rise to a large number of peptides, free amino acids, fatty acids and low-molecular-weight substances, which contribute to the flavour, aroma, texture and final quality of the cured meat products. Thus far, emerging technologies have not been used to enhance exopeptidase reactions in meat. However, high-power US could enhance mass transfer and, therefore, accelerate enzymatic activity to carry out a proteolytic degradation of peptides, which would contribute to an enhancement of the organoleptic characteristics of dry-cured products. In addition, the electrical stimulation of PEF and MEF, through the electroporation of cell membranes, would facilitate the extraction of proteins into the medium and intensify the enzymatic activity of exopeptidases in the hydrolysis of peptides. In addition, the application of SC-CO_2_ could improve the performance and activity of enzymes since it may cause changes in the conformational structure of the enzyme, favouring the enzymatic reactions. In addition, fermentation in sausages is carried out by endogenous microorganisms and microorganisms added as starter cultures (lactic acid bacteria, Staphylococcus, moulds and yeasts). In this case, the application of SC-CO_2_ could facilitate the mass transfer, intensifying the enzymatic reactions related to the starter cultures.

There is no literature that applies emerging technologies for the purposes of intensifying the enzymatic generation of peptides with the resulting beneficial health properties. Thus, the use of US could promote the breakdown of protein bonds and intensify the formation of peptides. Electrical stimulation (PEF and MEF) could cause the electroporation of cell membranes, enhancing the exit of proteins into the medium and accelerating the enzymatic reaction with both exogenous and endogenous enzymes.

As yet, emerging technologies have not been applied in conjunction with transglutaminase for the manufacturing of restructured meat products. In this regard, moderate-intensity US could enhance the binding between the enzyme and the substrate to develop new meat products. In addition, carrying out the enzymatic reaction in the SC-CO_2_ medium could cause an intensification of the process since temperature is not a limiting factor.

Finally, it could be concluded from the existing literature that most attributes defining meat and meat product quality, such as taste, texture or appearance, are closely related to the meat’s enzymatic reactions. Therefore, the use of emerging technologies, under controlled conditions, could allow an acceleration in the enzymatic processes and, therefore, an increase in the yield and quality of the meat products.

## Figures and Tables

**Table 1:** Influence of ultrasound (US) treatment on enzymatic reactions of non-meat and meat products.

Meat Products			
Sample	US Parameters (Power, Frequency, Temperature, pH, Time)	US Effects	Reference
Beef Semimembranosus muscles (lysosomal enzyme and β-glucuronidase)	10 W/cm^2^, 2.6 MHz, 2 consecutive periods of 15 s allowing a rest period of 2 min	Acceleration in the release of the lysosomal enzyme, and β-glucuronidase and weakening of the muscle structure	Got et al. [102]
Hen breast muscle (proteases)	12 W/cm^2^, 24 Hz, for 15 s	Increase in the degradation of muscular proteases; improvement in tenderisation	Xiong et al. [103]
Papain enzyme in chicken breast	135 W, 40 kHz, 10 min	Reduction in the hardness, improvement in tenderness	Lima et al. [104]
Leek extract proteases in beef	100 W, 37 kHz, 20 min	Increase in the proteolytic activity in beef, improvement in tenderness	Mehrabani et al. [105]
**Non-Meat Products**			
**Sample**	**US Parameters** **(Power, Frequency, Temperature, pH, Time)**	**US Effects**	**Reference**
Milk (β-Galactosidase from *Kluyveromyces marzianus*)	20 W and 20 kHz, 37 °C, pH 6.7, 30 min	Increase in the degree of lactose hydrolysis (14%) compared to the treatment without US	Şener et al. [91]
Sugar (invertase)	22 W/L, 25 Hz, 40 °C	Increase in the degree of carbohydrate hydrolysis (33%) with invertase compared to the treatment without US	de Souza Soares et al. [92]
Starch (glucoamylase)	7.2 W/mL, 22 kHz, 35 °C, 40 min	Acceleration in the degree of enzymatic hydrolysis, reducing the molecular weight of starch by 80.19% and increasing the solubility by 136.5%.	Wang et al. [93]
Soy sauce (protein)	126.4 W/cm^2^, 50 °C, for 20 min and 240 min	Acceleration in the degree of protein hydrolysis in 20 min by 4.98% and in 240 min by 8.48%.	Chen et al. [95]
Pectinase from *Aspergillus niger*	4.5 W/mL, 22 kHz, 20 °C for 10 min	Improvement in the reaction rate of the enzymatic hydrolysis process of citrus peel pectin (32.59%) to obtain galacturonic acid	Ma et al. [94]

**Table 2:** Influence of high-pressure processing (HPP) treatment on enzymatic reactions of non-meat and meat products.

Meat Products			
Sample	HPP Parameters (Pressure, Time, Temperature)	HPP Effects	Reference
Beef Biceps femoris muscle (metmyoglobin reductase)	130 MPa, 10 °C	Enhanced reduction of metmyoglobin to ferrous myoglobin	Jung et al. [120]
Serrano dry-cured ham (lipid oxidation, aminopeptidase and free amino acid activities)	600 MPa for 6 min	Increase in lipid peroxidation rates and decrease in the activity of all aminopeptidases studied	Rivas- Cañedo et al. [121]
**Non-Meat Products**			
**Sample**	**HPP Parameters** **(Pressure, Time, Temperature)**	**HPP Effects**	**Reference**
Goat cheese (casein)	400 MPa for 5 min	Acceleration in proteolysis process. The maturation was completed in 14 d with HPP, while normal maturation took 28 d	Saldo et al. [117]
β-lactoglobulin	200 MPa for 180 min	Complete hydrolysis of β-lactoglobulin was carried out by the enzyme thermolysin, observing that this enzyme only partially hydrolysed β-lactoglobulin in cow whey concentrate at atmospheric pressure and 25 °C.	Hayashi et al. [118]
Peptinase cocktail and black garlic juice	50 MPa for 240 min at 55 °C	Increase in the enzymatic activity of peptinase compared to treatment without HPP. The concentration of galacturonic acid released was higher than in the untreated black garlic juice.	Kim et al. [119]

**Table 3:** Influence of pulsed electric fields (PEF) treatment on enzymatic reactions of non-meat and meat products.

Meat Products			
Sample	PEF Parameters (Electric Field Strength, Pulse Width, Number of Pulses, Specific Energy, Time)	PEF Effects	Reference
Venison (calpain)	0.2 kV/cm, 1.93 kJ/kg, 20 μs and 0.5 kV/cm, 70.2 kJ/kg and 20 μs	Tendency towards increasing the calpain activity	Bhat et al. [137]
Beef Semimembranosus	10 kV, 90 Hz, 20 μs	Improvement in tenderness due to the phenomenon of electroporation; this allows Ca^2+^ release, which activates its dependent proteases, calpains	Carne et al. [138] Bekhit et al. [139] Warner et al. [124]
Beef Longissimus thoracis	0.2–0.6 kV/cm, 1–50 Hz, 20 μs	No influence on the activity of proteases; therefore, no improvement in meat tenderness	Faridnia et al. [140]
Chicken meat	3 kV/cm, 300 pulses of 20 μs	No influence on the activity of proteases; therefore, no improvement in meat tenderness	Arroyo et al. [141]
**Non-Meat Product**			
**Sample**	**PEF Parameters** **(Electric Field Strength, Pulse Width, Number of Pulses, Specific Energy, Time)**	**PEF Effects**	**Reference**
Papain	50 kV/cm with 500 pulses for 2 ms	70–10% Reduction in enzyme activity due to oxidation of cysteine amino acid residue located in the active site of papain	Yeom et al. [133]
Carrots (Ascobic acid oxidase)	0.2 to 1.2 kV/cm, 5 to 300 Hz, 20 μs	Carrots”, 3rd column “reduction in ascorbic acid oxidase activity up to 61%	Leong & Oey, [134]
Tomatoes (pectin methylesterase)	24 kV/cm,400 pulses of 0.02 ms pulse-width	Tomatoes”, 3rd column “reduction in tomato pectin methylesterase activity up to 94%	Giner et al. [135]
Apples and pears (polyphenoloxidase)	Apple extract: 24 kV/cm, 6 ms Pear extract: 22.3 kV/cm, 6 ms	Apples and pears”, 3rd column “reduction in polyphenoloxidase activities up to 38%	Giner et al. [136]

**Table 4:** Influence of moderate electric fields (MEF) treatment on enzymatic reactions of non-meat and meat products.

Meat Products			
Sample	MEF Parameters (Electric Field Strength, Frequency, Temperature, Time)	MEF Effects	Reference
Beef	10–600 V, 50 Hz, 2–100 pulses/s, 5- 120 s	Acceleration in post mortem glycolysis, improving tenderization	Chrystall et al. [155]
Beef	550 V, 60 Hz, 1 s pulse and 0.5 s rest, during 120 s	Electroporation of the muscle cell membrane, affecting the release of Ca^2+^ and the activation of calpain	Ducastaing et al. [156]
**Non-Meat Products**			
**Sample**	**MEF Parameters** **(Electric Field Strength, Frequency, Temperature, Time)**	**MEF Effects**	**Reference**
Corn starch	0–10 V/cm, 50 Hz, 30 min	Hydrolysis of corn starch, activating glucoamylase	Li et al. [149].
Tomato homogenate	8 V/cm, 60 Hz sinusoidal wave, reaching 80 °C starting at 65 °C	Activation of the pectin methyl esterase (PME)	Samaranayake et al. [150]
Sugarcane juice	3.57 V/cm, 98 °C for 12 min	Inactivation of the enzymes peroxidase and polyphenol oxidase	Brochier et al. [152]
Pea puree	50 V/cm, 100 °C for 54 s	Inactivation of the enzyme peroxidase	Icier et al. [153]

**Table 5:** Influence of supercritical carbon dioxide (SC-CO_2_) treatment on enzymatic reactions of non-meat and meat products.

Non-Meat Products			
Sample	SC-CO_2_ Parameters (Pressure, Temperature, Time, Flow)	SC-CO_2_ Effects	Reference
Immobilization of cellulase in a system consisting of enzyme aggregates cross-linked (CLEA) with solvents and glutaraldehyde.	10 MPa, 50 °C, 3 h	Increase in the enzyme activity by 57% compared to the enzyme not treated with CO_2_	Hojnik Podrepšek et al. [161]
α-amylase	240 bar, 41 °C, 50 min, 4 g/min CO_2_ flow	Increase in the enzyme activity (67.7%) compared to the enzyme not treated with CO_2_	Senyay-Oncel & Yesil-Celiktas [162]

## Data Availability

Data is contained within the article.
